# TLR4 single nucleotide polymorphisms (SNPs) associated with *Salmonella* shedding in pigs

**DOI:** 10.1007/s13353-014-0199-8

**Published:** 2014-02-25

**Authors:** Jalusa Deon Kich, Jolita Janutenaite Uthe, Magda Vieira Benavides, Maurício Egídio Cantão, Ricardo Zanella, Christopher Keith Tuggle, Shawn Michelle Dunkin Bearson

**Affiliations:** 1 USDA/ARS/National Animal Disease Center, 1920 Dayton Ave, Ames, IA USA; 2 Embrapa Swine and Poultry, Br 153, Km 110, Concórdia, SC Brazil; 3 Department of Animal Science, Iowa State University, 2255 Kildee Hall, Ames, IA USA; 4 Embrapa LabEx USA/USDA, Beltsville Agriculture Research Center, Beltsville, MD USA; 5 Embrapa Swine and Poultry BJT/CNPq, Br 153, Km110, Concórdia, SC Brazil

**Keywords:** *Salmonella*, Single nucleotide polymorphisms (SNPs), Swine, *TLR4*

## Abstract

Toll-like receptor 4 (TLR4) is a key factor in the innate immune recognition of lipopolysaccharide (LPS) from Gram-negative bacteria. Previous studies from our group identified differences in the expression profile of *TLR4* and genes affected by the TLR4 signaling pathway among pigs that shed varying levels of *Salmonella*, a Gram-negative bacterium. Therefore, genetic variation in this gene may be involved with the host’s immune response to bacterial infections. The current study screened for single nucleotide polymorphisms (SNPs) in the *TLR4* gene and tested their association with *Salmonella* fecal shedding. Pigs (*n* = 117) were intranasally challenged at 7 weeks of age with 1 × 10^9^ CFU of *S*. Typhimurium χ4232 and were classified as low or persistent *Salmonella* shedders based on the levels of *Salmonella* being excreted in fecal material. *Salmonella* fecal shedding was determined by quantitative bacteriology on days 2, 7, 14, and 20/21 post exposure, and the cumulative levels of *Salmonella* were calculated to identify the low (*n* = 20) and persistent (*n* = 20) *Salmonella* shedder pigs. From those 40 animals, the *TLR4* region was sequenced, and 18 single nucleotide polymorphisms (SNPs) in *TLR4* were identified. Twelve SNPs have been previously described and six are novel SNPs of which five are in the 5′ untranslated region and one is in intron 2. Single marker association test identified 13 SNPs associated with the qualitative trait of *Salmonella* fecal shedding, and seven of those SNPs were also associated with a quantitative measurement of fecal shedding (*P* < 0.05). Using a stepwise regression process, a haplotype composed of SNPs *rs80787918* and *rs80907449* (*P* ≤ 4.0 × 10^−3^) spanning a region of 4.9 Kb was identified, thereby providing additional information of the influence of those SNPs on *Salmonella* fecal shedding in pigs.

**Electronic supplementary material:**

The online version of this article (doi:10.1007/s13353-014-0199-8) contains supplementary material, which is available to authorized users.

## Introduction

*Salmonella* is a widespread foodborne pathogen with the ability to adapt to different environments, consequently creating significant challenges to food-producing industries in controlling this pathogen in food chain products. Swine (*Sus scrofa*) are an important reservoir of *Salmonella* because colonization and shedding of this bacterium occurs within asymptomatic pigs, imposing elevated risks to public and animal health. Thus, diverse intervention strategies are needed to control the transmission of *Salmonella* from pig products to humans and to the environment.

In bacterial infections, the severity of infection is impacted by the pathogenicity of the microorganism and its interaction with the host immune defense system (Zanella et al. 2011). Toll-like receptor 4 (TLR4) is a well-characterized gram-negative bacterial lipopolysaccharide (LPS) recognition receptor and a host inflammatory response activator well conserved among animal species (Noreen et al. 2012; Yang et al. 2012). Schröder and Schumann (Schröder and Schumann 2005) suggested that mutations in the *TLR4* regions involved with pathogen recognition and transduction signaling may affect host susceptibility to infection. Polymorphisms in the *TLR4* gene have been associated with different infectious diseases in humans, such as meningitis and tuberculosis, as well as some types of cancers (Noreen et al. 2012) and with infection and disease in cattle, chicken and pigs (Yang et al. 2012; Kataria et al. 2011; Leveque et al. 2003).

In swine, *TLR4* is located on *Sus scrofa 1* (SSC1) V10.2 (289,776,058 bp to 289,785,087 bp). Thomas et al. (2006) identified the genomic structure of porcine *TLR4*, and Shinkai et al. (2006) described the distribution of SNPs for five *TLR*s in pigs. Specifically for *TLR4*, 13 SNPs were widely distributed in 11 pig breeds, and of those, seven were non-synonymous. Thirty four SNPs were identified in *TLR4* using pigs representing European commercial breeds and some traditional breeds (*n* = 259), and of these, 17 SNPs were located in the non-coding region and 17 SNPS were found in the coding region (Palermo et al. 2000). Furthermore, polymorphisms in the *TLR4* gene have been identified as potential genetic markers for disease susceptibility in pigs (Uenishi and Shinkay 2009). Our collaborative group has reported up-regulation of *TLR4* and its target genes in pigs challenged with *Salmonella enterica* serovar Typhimurium (Huang et al. 2011). Therefore, to determine if *TLR4* is a possible candidate gene associated with *Salmonella* shedding, we first, identified SNPs in the *TLR4* gene of our previously described low and persistent shedder pig populations (Huang et al. 2011; Uthe et al. 2009, 2011). Second, we investigated associations of the *TLR4* SNPs with *Salmonella* shedding status. Selecting for pigs with reduced *Salmonella* fecal shedding would decrease environmental contamination and lower pathogen transmission to other animals and humans; thus, identification of loci in *TLR4* associated with *Salmonella* fecal shedding is the focus of this study.

## Material and methods

All procedures involving animals in the NADC-40 and NADC-77 populations were approved by the USDA, ARS, NADC Animal Care and Use Committee. Briefly, all the pigs used in this study were intranasally challenged at 7 weeks of age with 1 × 10^9^ CFU of *S.* Typhimurium χ4232 as previously described (Huang et al. 2011; Uthe et al. 2009). At days 2, 7, 14, and 20/21 post-inoculation (pi), *Salmonella* fecal shedding was quantified using a standard bacteriological test previously described (Uthe et al. 2009).

Of the initial 117 animals, 40 (*n* = 40) pigs were chosen based on their extreme fecal culture status; quantitative classification of the phenotype was scored based on cumulative *Salmonella* fecal shedding on days 2, 7, 14, and 20/21 pi (Huang et al. 2011). Genomic DNA was extracted from blood samples and purified as previously described (Uthe et al. 2011). Nine sets of primers were designed based on the Ensembl gene sequence for ENSSSCG00000005503 using Beacon Designer (Table 1). Primers were selected to cover all exons (*n* = 3) of *TLR4* including a 713 bp upstream region and a 1225 bp downstream region (SSC1: 289,775,345 bp–289,786,312 bp Ensembl genome build 10.2). The PCR mix for each reaction contained 16.75 μl dH_2_O, 1.25 μl each primer (10 μM), 2 μl of dNTPs, 2.5 μl 10X Buffer with MgCl_2,_ 0.25 μl Platinum Taq DNA Polymerase High Fidelity (Invitrogen Grand Island, NY, USA) and 1 μl of 10 ng/μl pig DNA. DNA samples were amplified using the MJ Research PTC-200 PCR thermal cycler (BioRad Laboratories, Hercules, CA). The PCR reaction was performed as follows: 94 °C for 2 min, 30 cycles of 94 °C for 30 s, 58 °C for 30 s, 72 °C for 1 min and a final step of 72 °C for 7 min. PCR products were visualized by agarose gel electrophoresis to confirm a single correct size product and purified using MinElute 96 UF PCR purification kit (Qiagen) prior to DNA sequencing using an AB 3730xl DNA Analyzer (Applied Biosystems) at Iowa State University, Ames, IA.

**Table 1:** Identified SNPs and position in the *TLR4* gene of *Salmonella* low and persistent shedder pigs

				Single marker association (*P*-value)		GenBank accession number for the SNP	Location in Sus Scrofa genome (bp)	Amino acid	
N	Location	Primers	SNP designation	Qualitative	Quantitative			Site	Amino acid
1	5′Upstream	5′gaaccatgcagtagaacagg 3′ctggaagtctgtagtcaagg	^1^5′U:A-1082G#	0.033	0.064	No	SSC1:289,774,983	–	–
2			^1^5′U:T-1019C#	0.033	0.064	No	SSC1:289,775,046	–	–
3			^1^5′U:C-984T#	0.033	0.064	No	SSC1:289,775,081	–	–
4		5′cacaagaaggaagagatagc 3′ caccaagggaagctctagg	^1^5′U:C-522T#	0.133	0.244	No	SSC1:289,775,543		
5			^1^5′U:G-400A	0.363	0.550	rs80830544	SSC1:289,775,665		
6			^1^5′U:G-75C#	0.025	0.056	No	SSC1:289,775,979	–	–
7	Intron 2	5′acagaagattggatggaagga 3′ gagataagaaagctgagacc	^2^2I:A232C	0.004	0.029	rs80881287	SSC1:289,780,226	–	–
8			^2^2I:C298T**	0.002	0.013	rs80787918	SSC1:289,780,292	–	–
9	Intron 2	5′cctcacttgatatgtttgcc 3′gttcctccaggacagatttg	^2^2I:C2567T#	0.001	0.025	No	SSC1:289,782,761	–	–
10	Exon 3		^3^C318A	0.003	0.037	rs80923358	SSC1:289,782,834	–	–
11			^3^G417A	0.003	0.037	rs80951861	SSC1:289,782,933	–	–
12			^3^T611A*	0.007	0.054	rs80811682	SSC1:289,783,127	204	L/H
13	Exon 3	5′attcaaggtctggctggttc 3′ tgaagacatcaggaagcaag	^3^G826A*	0.285	0.514	Shinkai et al. (2006)	SSC1:289,783,342	276	V/I
14			^3^G960A	0.064	0.105	rs80981701	SSC1:289,783,476	–	–
15			^3^G962A*	0.034	0.046	rs80955017	SSC1:289,783,478	321	R/H
16			^3^C1027A*	0.176	0.231	rs80894552	SSC1:289,783,543	343	Q/K
	Exon 3	5′acatccacgttgtcttccg 3′cagttcattcctcacccag	–					–	–
17	Exon 3	5′cttcctcctggtatctgtgg 3′ggcagtcctgtgtatctcg	^3^G2397A	0.025	0.056	rs80834103	SSC1:289,784,913	–	–
18	3′Downstream	5′actcccaacgtgtcccttg 3′ccaagaagtgccactttcaac	^4^3′D:C208T**	0.002	0.011	rs80907449	SSC1:289,785,250	–	–

^1^to first codon of exon 1; ^2^position in intron 2; ^3^position in coding region; ^4^position in 3′UTR downstream of last codon; *non-synonymous SNPs; **haplotype components; # novel SNPs

Sequences were analyzed and polymorphisms were identified using Phred/Phrap/Consed/PolyPhred software (Nickerson et al. 1997; Ewing et al. 1998; Gordon et al. 1998; [internet] http://www phrap org). Genotypic data were assessed for quality before the association analysis. SNPs were also assessed for quality prior to the association analysis. SNPs were removed if the minor allele frequency (MAF) was less than 10 %, if the SNPs failed to genotype in more than 10 % of the samples, or if the SNPs failed the Hardy–Weinberg equilibrium (*P* < 0.001). No animals or SNPs were removed from the analysis due to genotypic quality. Statistical analyses were conducted within PLINK and R statistical environment (version 1.07, (Purcell et al. 2007)).

A chi-squared test (χ^2^) was used to test associations of SNPs located in *TLR4* and the qualitative measurement of *Salmonella* shedding (persistent versus low). The Wald test was used to verify associations with *Salmonella* shedding as a quantitative trait. A significance threshold for the association analysis was set to *P* ≤ 0.05. Following the single marker association test, a haplotype test was conducted within PLINK to identify if a haplotype was more informative than a single SNP. First, an omnibus association test was performed to identify the overall association of the haplotype with the qualitative measurement of *Salmonella* shedding. If an association was identified, a haplotype-specific test was performed to identify which combination of the alleles provided the strongest evidence for an association with *Salmonella* shedding in swine. Following haplotype construction, a stepwise regression using a backward-elimination process was performed to identify the effect of each associated SNP in relationship to the haplotype; in this test, all associated SNPs were included and excluded individually from the analysis, and the association of the haplotype was tested each time using PLINK.

## Results and discussion

Huang et al. (2011) identified the *TLR4*-dependent set of genes (TLR4 regulon) as a major inducer of the transcriptional response in *Salmonella* persistently shedding pigs, and this TLR4 regulon was not significantly affected in the low shedding pigs. Thus, *TLR4* is considered a potential candidate to analyze the association of genetic polymorphisms with the diverse phenotypic patterns of *Salmonella* shedding in swine.

**Fig. 1:**
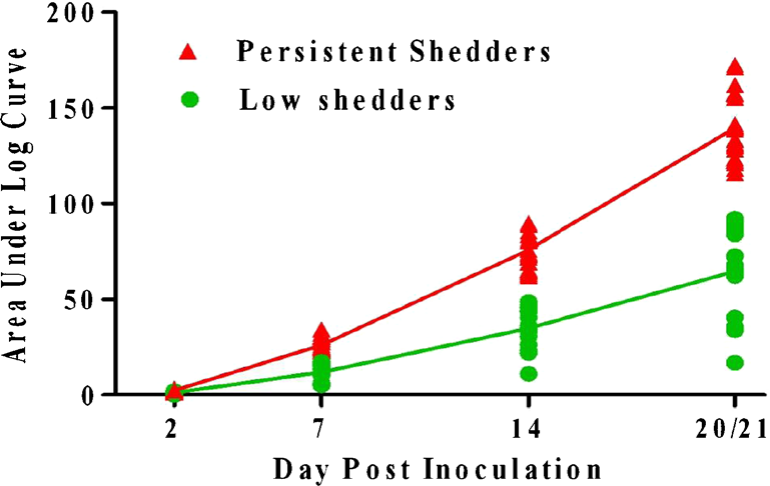
Area under the log curve illustrating the log of cumulative colony forming units (CFU). Quantitative bacteriology of *Salmonella* shedding in swine fecal samples was performed at day 2, 7, 14, and 20/21 days post-challenge with *Salmonella enterica* serovar Typhimurium, and CFU were determined

In this study, two swine populations were investigated, NADC-40 and NADC-77 (Uthe et al. 2011), each population with 10 low and 10 persistent *Salmonella* shedding animals (Fig. 1). For the quantitative measurement of *Salmonella* shedding per pig, a cumulative measurement taken within days 2, 7, 14, and 20/21 pi was calculated (Huang et al. 2011). Sequencing analysis of those 40 (*n* = 40) animals identified 18 SNPs; 12 were previously described in the literature and/or annotated in GenBank and six are novel SNPs (Table 1). Five of the novel SNPs are located within the 5′untranslated region (UTR) and one is within intron 2.

The swine *TLR4* gene (9030 bp/SGSC Sscrofa10.2/susScr3) is composed of three exons (93, 167, and 2266 bp). Taking together these results and the literature, 50 SNPs have been identified in *TLR4*, with 22 SNPs located in the coding regions (Thomas et al. 2006; Shinkai et al. 2006; Palermo et al. 2000; Pan et al. 2011; Bao et al. 2011; Shinkai et al. 2012). Of these 22 SNPs, nine are non-synonymous and located on exon 3. Our investigation identified four of those nine non-synonymous SNPs in exon 3 segregating in the NADC-40 and NADC-77 pig populations. Four segregating synonymous SNPs were also detected in exon 3.

Of the 18 SNPs identified in the two pig populations, 13 (*n* = 13) SNPs were associated (*P* ≤ 0.05) with *Salmonella* shedding as a qualitative phenotype using a Chi-squared test; of those 13 SNPs, seven were also associated with *Salmonella* shedding as a quantitative phenotype using a Wald statistical test (Table 1). Using a haplotype construction and the backward-elimination process, the most significant haplotype for both measurements of *Salmonella* shedding, qualitative (*P* ≤ 7.9 × 10^−4^) and quantitative (*P* ≤ 4.0 × 10^−3^) (Table 2) comprised a region of 4.9 Kb composed of SNPS, *rs80787918* (SNP8) and *rs80907449* (SNP18) (r^2^ = 0.902) located at SSC1:289,780,292 bp and SSC1:289,785,250 bp, respectively (Table 1).

**Table 2:** Haplotypes frequency (SNPs *rs80787918* and *rs80907449*) and associations with qualitative and quantitative phenotypes of *Salmonella* shedding

	Haplotype frequency		Qualitative *P* value	Quantitative *P* value
Haplotype	Persistent shedders	Low shedders		
CC	0.675	0.3	0.00079	0.004201
TC	0	0.025	0.3143	0.1054
CT	0	0.025	0.3143	0.1445
TT	0.325	0.65	0.00334	0.02912

Four SNPs, *rs80811682 (SNP12), SNP13, rs80955017 (SNP15)*, and *rs80894552 (SNP16),* located on exon 3 of *TLR4* gene are non-synonymous mutations and they are positioned between markers *rs80787918* (*SNP8*) and *rs80907449* (*SNP18*). When the additive effect of those markers was tested within the haplotype constructed with markers *rs80787918* (*SNP8*) and *rs80907449* (*SNP18*), we did not observe any improvement in the association test. However, analyzing together the markers *rs80787918* (*SNP8*)*, rs80811682* (*SNP12*)*, SNP13, rs80955017* (*SNP15*)*, rs80894552* (*SNP16*) *and rs80907449* (*SNP18*), the haplotype composed of alleles (CTGGCC) was found in higher frequency (65 %) in persistent shedders than 31 % in low shedders pigs (*P* < 0.003). Possibly, the addition of more markers in the haplotype is being penalized by the increased number of degrees of freedom and reduced number of samples per each class affecting the significance of our association results. To overcome this problem we tested multiple haplotypes with fewer markers per test. When markers *SNP13* and *rs80894552 (SNP16),* which were not significant in the single marker association test, were removed from the haplotype, the significance improved to *P* < 0.001. The haplotype composed of alleles CTGC was found in 65 % of the high shedders and 30 % of low shedders and the haplotype (TAAT) was found in 20 % of the high shedders and 41 % of the low shedders (*P* < 0.04). Finally, a specific haplotype (CTC) constructed with markers *rs80787918* (*SNP8*)*, rs80811682* (*SNP12*) and *rs80907449* (*SNP18*), was observed in 65 % of the persistent shedders and 30 % on the low shedders pigs (*P* < 0.001). The opposite haplotype (TAT) was observed in 27.5 % of the persistent shedders and in 59.4 % of the low shedders pigs (*P* < 0.003).

A trend was observed between haplotypes constructed with markers: *rs80787918 (SNP8), SNP13 and rs80907449* (*SNP18*) (CGC); *rs80787918* (*SNP8*), *rs80955017* (*SNP15*) *and rs80907449* (*SNP18*) (CGC); *rs80787918* (*SNP8*)*, rs80894552* (*SNP16*) *and rs80907449* (*SNP18*) (CCC), where they were observed in 67.5 % of the persistent shedders and 30 % of low shedder pigs.

Haplotype CC of SNPS *rs80787918* (*SNP8*) and *rs80907449 (SNP18)* was identified in higher frequency in persistent shedding pigs (67.5 %: *n* = 14) compared to low shedding pigs (30 %; *n* = 6); furthermore, the frequency of haplotype TT in low shedding pigs (65 %; *n* = 13) was greater when compared to persistent shedding pigs (32.5 %; *n* = 6). No animals from the persistent shedding group were identified with the haplotype TC or CT, while it was observed in low frequency in the low shedding group (2.5 %). Together, these results suggest that the region located between markers *rs80787918* and *rs80907449,* more specifically on exon 3, is possibly harboring the causative mutation for *Salmonella* colonization and shedding variation in swine.

## Conclusion

The results from this study support the concept that TLR4 is an important modulator associated with the porcine response to *Salmonella* infection in swine. Particularly interesting is that the haplotype with the highest significant association to the shedding phenotypes was found most often (∼65 %) in the persistent shedder pigs than in low shedder pigs. Genetic variation in molecular functional regions, such as a ligand recognition site, can alter host resistance/susceptibility to specific pathogens (Uenishi et al. 2011). Furthermore, synonymous mutations in a gene can play a significant role in transcriptional regulation (Sauna and Kimchi-Sarfaty 2011; Sato et al. 2012). Thus, similar to Shinkai et al. (2011) who demonstrated polymorphisms in *TLR5* and *TLR2* alter the cellular response to *S.* Choleraesuis, our results highlight the importance of linking genetic variations that may influence the molecular function of a key transcriptional regulator (TLR4) with *Salmonella* shedding in swine.

## Electronic supplementary material

Below is the link to the electronic supplementary material.

ESM 1 (DOCX 32 kb)

## References

[CR1] Bao WB, Ye L, Pan ZY (2011). Analysis of polymorphisms in the porcine TLR4 gene and its expression related to *Escherichia coli* F18 infection. Czech J Anim Sci.

[CR2] Ewing B, Hillier L, Wendl MC, Green P (1998). Base-calling of automated sequencer traces using phred. I. Accuracy assessment. Genome Res.

[CR3] Gordon D, Abajian C, Green P (1998). Consed: a graphical tool for sequence finishing. Genome Res.

[CR4] Huang T, Uthe JJ, Bearson SMD (2011). Distinct peripheral blood RNA responses to *Salmonella* in pigs differing in *Salmonella* shedding levels: intersection in IFNG, TLR and miRNA pathways. PloS ONE.

[CR5] [internet] http://www.phrap.org

[CR6] Kataria RS, Tait RG, Kumar D (2011). Association of toll-like receptor four single nucleotide polymorphisms with incidence of infectious bovine keratoconjunctivitis (IBK) in cattle. Immunogenetics.

[CR7] Leveque G, Forgetta V, Morroll S (2003). Allelic variation in TLR4 is linked to susceptibility to *Salmonella enterica* serovar Typhimurium infection in chickens. Infect Immun.

[CR8] Nickerson DA, Tobe VO, Taylor SL (1997). PolyPhred: automating the detection and genotyping of single nucleotide substitutions using fluorescence-based resequencing. Nucleic Acids Res.

[CR9] Noreen M, Shah MAA, Mall SM (2012). TLRT4 polymorphisms and diseases susceptibility. Inflamm Res.

[CR10] Palermo S, Capra E, Torremorell M (2000). Toll-like receptor 4 genetic diversity among pig populations. Anim Genet.

[CR11] Pan ZY, Ye L, Zhu J (2011). Isolation of new alleles of the swine TLR4 gene and analysis of its genetic variation. Yi Chuan.

[CR12] Purcell S, Neale B, Todd-Brown K (2007). PLINK: a tool set for whole-genome association and population-based linkage analyses. Am J Hum Genet.

[CR13] Sato K, Yoshimura A, Kaneko T (2012). A single nucleotide polymorphism in 3′-untraslated region contribute to the regulation of Toll-like receptor 4 translation. J Biol Chem.

[CR14] Sauna ZE, Kimchi-Sarfaty C (2011). Undesrtanding the contribuition of synonymous mutations to human diseases. Nat Rev Genet.

[CR15] Schröder NWJ, Schumann RR (2005). Single nucleotide polymorphisms of Toll-like receptors and susceptibility to infectious disease. Lancet Infect Dis.

[CR16] Shinkai H, Tanaka M, Morozumi T (2006). Biased distribuition of single nucleotide polymorphisms (SNPs) in porcine Toll-like receptor 1 (TLR1), TLR2, TLR4, TLR5 and TLR6 genes. Immunogenetics.

[CR17] Shinkai H, Suzuky R, Akiba M (2011). Porcine Toll-like receptors: recognition of *Salmonella enterica* serovar Choleraesuis and influence of polymorphisms. Mol Immunol.

[CR18] Shinkai H, Okumura N, Suzuki R (2012). Toll-like receptor 4 polymorphism impairing lipopolysaccharide signaling in *Sus scrofa*, and its restricted distribution among Japanese wild boar population. DNA Cell Biol.

[CR19] Thomas AV, Broes AD, Vandegaart HF (2006). Genomic structure, promoter analysis and expression of the porcine (*Sus scrofa*) TLR4 gene. Mol Immunol.

[CR20] Uenishi H, Shinkay H (2009). Porcine Toll-like receptors: the front line of pathogen monitoring and possible implications for disease resistance. Dev Comp Immunol.

[CR21] Uenishi H, Shinkai H, Morozumi T (2011). Genomic survey of polymorphisms in pattern recognition receptors and their possible relationship to infection in pigs. Vet Immunol Immunopathol.

[CR22] Uthe JJ, Wang Y, Qu L (2009). Correlating blood immune parameters and CCT7 genetic variant with the shedding of *Salmonella enterica* serovar Typhimurium in swine. Vet Microbiol.

[CR23] Uthe JJ, Qu L, Couture O (2011). Use of bioinformatics SNP predictions in differentially expressed genes to find SNPs associated with *Salmonella* colonization in swine. J Anim Breed Genet.

[CR24] Yang XQ, Murani E, Ponsuksili S (2012). Association of TLR4 polymorphism with cytokine expression level and pulmonary lesion in pigs. Mol Biol Rep.

[CR25] Zanella R, Settles ML, McKay SD (2011). Identification of loci associated with tolerance to Johne’s disease in Holstein cattle. Anim Genet.

