# Supplementary material for: TLR4 single nucleotide polymorphisms (SNPs) associated with Salmonella shedding in pigs
Source: J Appl Genet. 2014 Feb 25;55(2):267–71. doi: 10.1007/s13353-014-0199-8 (PMC3990860; doi:10.1007/s13353-014-0199-8)
Supplement: Supplementary file 1 — (DOCX 32 kb) [file 13353_2014_199_MOESM1_ESM.docx]

Haplotype construction analysis

| Haplotype Number | Haplotype | Haplotype Frequency  Persistent Shedders | Haplotype Frequency  Low Shedders | Qualitative P-Value | Quantitative P-Value | SNPS |
| --- | --- | --- | --- | --- | --- | --- |
| H1 | C | 0,6750 | 0,3250 | 0,0017 | 0,0132 | snp8 |
| H1 | T | 0,3250 | 0,6750 | 0,0017 | 0,0132 | snp8 |
| H2 | C | 0,6750 | 0,3250 | 0,0017 | 0,0107 | snp18 |
| H2 | T | 0,3250 | 0,6750 | 0,0017 | 0,0107 | snp18 |
| H3 | TTAAA | 0,2500 | 0,6000 | 0,0015 | 0,0256 | snp8\|snp9\|snp10\|snp11\|snp12 |
| H3 | TCAAA | 0,0250 | 0,0000 | 0,3143 | 0,3922 | snp8\|snp9\|snp10\|snp11\|snp12 |
| H3 | CCCGA | 0,0250 | 0,0000 | 0,3143 | 0,5436 | snp8\|snp9\|snp10\|snp11\|snp12 |
| H3 | CCCGT | 0,6500 | 0,3250 | 0,0036 | 0,0214 | snp8\|snp9\|snp10\|snp11\|snp12 |
| H3 | TCCGT | 0,0500 | 0,0750 | 0,6442 | 0,5996 | snp8\|snp9\|snp10\|snp11\|snp12 |
| H4 | TTAAAA | 0,0500 | 0,1625 | 0,1025 | 0,1234 | snp8\|snp9\|snp10\|snp11\|snp12\|snp13 |
| H4 | TCCGTA | 0,0500 | 0,0250 | 0,5562 | 0,2709 | snp8\|snp9\|snp10\|snp11\|snp12\|snp13 |
| H4 | TTAAAG | 0,2000 | 0,4375 | 0,0227 | 0,0451 | snp8\|snp9\|snp10\|snp11\|snp12\|snp13 |
| H4 | TCAAAG | 0,0250 | 0,0000 | 0,3143 | 0,3922 | snp8\|snp9\|snp10\|snp11\|snp12\|snp13 |
| H4 | CCCGAG | 0,0250 | 0,0000 | 0,3143 | 0,5436 | snp8\|snp9\|snp10\|snp11\|snp12\|snp13 |
| H4 | CCCGTG | 0,6500 | 0,3250 | 0,0036 | 0,0214 | snp8\|snp9\|snp10\|snp11\|snp12\|snp13 |
| H4 | TCCGTG | 0,0000 | 0,0500 | 0,1521 | 0,1054 | snp8\|snp9\|snp10\|snp11\|snp12\|snp13 |
| H5 | CCCGTGG | 0,6500 | 0,3250 | 0,0036 | 0,0214 | snp8\|snp9\|snp10\|snp11\|snp12\|snp13\|snp14 |
| H5 | TTAAAGA | 0,2000 | 0,4375 | 0,0227 | 0,0451 | snp8\|snp9\|snp10\|snp11\|snp12\|snp13\|snp14 |
| H5 | TCAAAGA | 0,0250 | 0,0000 | 0,3143 | 0,3922 | snp8\|snp9\|snp10\|snp11\|snp12\|snp13\|snp14 |
| H5 | TCCGTAG | 0,0500 | 0,0250 | 0,5562 | 0,2709 | snp8\|snp9\|snp10\|snp11\|snp12\|snp13\|snp14 |
| H5 | TTAAAAG | 0,0500 | 0,1625 | 0,1025 | 0,1234 | snp8\|snp9\|snp10\|snp11\|snp12\|snp13\|snp14 |
| H5 | TCCGTGG | 0,0000 | 0,0500 | 0,1521 | 0,1054 | snp8\|snp9\|snp10\|snp11\|snp12\|snp13\|snp14 |
| H5 | CCCGAGG | 0,0250 | 0,0000 | 0,3143 | 0,5436 | snp8\|snp9\|snp10\|snp11\|snp12\|snp13\|snp14 |
| H6 | CCCGTGGG | 0,6500 | 0,3250 | 0,0036 | 0,0214 | snp8\|snp9\|snp10\|snp11\|snp12\|snp13\|snp14\|snp15 |
| H6 | TTAAAGAA | 0,1750 | 0,4087 | 0,0215 | 0,0549 | snp8\|snp9\|snp10\|snp11\|snp12\|snp13\|snp14\|snp15 |
| H6 | TCAAAGAA | 0,0250 | 0,0000 | 0,3143 | 0,3922 | snp8\|snp9\|snp10\|snp11\|snp12\|snp13\|snp14\|snp15 |
| H6 | TTAAAGAG | 0,0250 | 0,0298 | 0,8949 | 0,8249 | snp8\|snp9\|snp10\|snp11\|snp12\|snp13\|snp14\|snp15 |
| H6 | TCCGTAGG | 0,0500 | 0,0250 | 0,5562 | 0,2709 | snp8\|snp9\|snp10\|snp11\|snp12\|snp13\|snp14\|snp15 |
| H6 | TTAAAAGG | 0,0500 | 0,1615 | 0,1050 | 0,1265 | snp8\|snp9\|snp10\|snp11\|snp12\|snp13\|snp14\|snp15 |
| H6 | TCCGTGGG | 0,0000 | 0,0250 | 0,3143 | 0,1054 | snp8\|snp9\|snp10\|snp11\|snp12\|snp13\|snp14\|snp15 |
| H6 | TCCGTGGA | 0,0000 | 0,0250 | 0,3143 | 0,1054 | snp8\|snp9\|snp10\|snp11\|snp12\|snp13\|snp14\|snp15 |
| H6 | CCCGAGGG | 0,0250 | 0,0000 | 0,3143 | 0,5436 | snp8\|snp9\|snp10\|snp11\|snp12\|snp13\|snp14\|snp15 |
| H7 | CCCGTGGGC | 0,6500 | 0,3250 | 0,0036 | 0,0214 | snp8\|snp9\|snp10\|snp11\|snp12\|snp13\|snp14\|snp15\|snp16 |
| H7 | TTAAAGAAC | 0,1750 | 0,4087 | 0,0215 | 0,0549 | snp8\|snp9\|snp10\|snp11\|snp12\|snp13\|snp14\|snp15\|snp16 |
| H7 | TCAAAGAAC | 0,0250 | 0,0000 | 0,3143 | 0,3922 | snp8\|snp9\|snp10\|snp11\|snp12\|snp13\|snp14\|snp15\|snp16 |
| H7 | TTAAAGAGC | 0,0250 | 0,0298 | 0,8949 | 0,8249 | snp8\|snp9\|snp10\|snp11\|snp12\|snp13\|snp14\|snp15\|snp16 |
| H7 | TCCGTAGGA | 0,0500 | 0,0250 | 0,5562 | 0,2709 | snp8\|snp9\|snp10\|snp11\|snp12\|snp13\|snp14\|snp15\|snp16 |
| H7 | TTAAAAGGA | 0,0500 | 0,1615 | 0,1050 | 0,1265 | snp8\|snp9\|snp10\|snp11\|snp12\|snp13\|snp14\|snp15\|snp16 |
| H7 | TCCGTGGAA | 0,0000 | 0,0250 | 0,3143 | 0,1054 | snp8\|snp9\|snp10\|snp11\|snp12\|snp13\|snp14\|snp15\|snp16 |
| H7 | TCCGTGGGC | 0,0000 | 0,0250 | 0,3143 | 0,1054 | snp8\|snp9\|snp10\|snp11\|snp12\|snp13\|snp14\|snp15\|snp16 |
| H7 | CCCGAGGGC | 0,0250 | 0,0000 | 0,3143 | 0,5436 | snp8\|snp9\|snp10\|snp11\|snp12\|snp13\|snp14\|snp15\|snp16 |
| H8 | CCCGTGGGCG | 0,6500 | 0,3250 | 0,0036 | 0,0214 | snp8\|snp9\|snp10\|snp11\|snp12\|snp13\|snp14\|snp15\|snp16\|snp17 |
| H8 | TTAAAGAACA | 0,1750 | 0,4181 | 0,0173 | 0,0492 | snp8\|snp9\|snp10\|snp11\|snp12\|snp13\|snp14\|snp15\|snp16\|snp17 |
| H8 | TCAAAGAACA | 0,0250 | 0,0000 | 0,3143 | 0,3922 | snp8\|snp9\|snp10\|snp11\|snp12\|snp13\|snp14\|snp15\|snp16\|snp17 |
| H8 | TTAAAGAGCA | 0,0250 | 0,0319 | 0,8520 | 0,7833 | snp8\|snp9\|snp10\|snp11\|snp12\|snp13\|snp14\|snp15\|snp16\|snp17 |
| H8 | TCCGTAGGAG | 0,0500 | 0,0250 | 0,5562 | 0,2709 | snp8\|snp9\|snp10\|snp11\|snp12\|snp13\|snp14\|snp15\|snp16\|snp17 |
| H8 | TTAAAAGGAG | 0,0500 | 0,1500 | 0,1360 | 0,1747 | snp8\|snp9\|snp10\|snp11\|snp12\|snp13\|snp14\|snp15\|snp16\|snp17 |
| H8 | TCCGTGGAAG | 0,0000 | 0,0250 | 0,3143 | 0,1054 | snp8\|snp9\|snp10\|snp11\|snp12\|snp13\|snp14\|snp15\|snp16\|snp17 |
| H8 | TCCGTGGGCG | 0,0000 | 0,0250 | 0,3143 | 0,1054 | snp8\|snp9\|snp10\|snp11\|snp12\|snp13\|snp14\|snp15\|snp16\|snp17 |
| H8 | CCCGAGGGCG | 0,0250 | 0,0000 | 0,3143 | 0,5436 | snp8\|snp9\|snp10\|snp11\|snp12\|snp13\|snp14\|snp15\|snp16\|snp17 |
| H9 | CCCGTGGGCGT | 0,0000 | 0,0250 | 0,3143 | 0,1445 | snp8\|snp9\|snp10\|snp11\|snp12\|snp13\|snp14\|snp15\|snp16\|snp17\|snp18 |
| H9 | CCCGTGGGCGC | 0,6500 | 0,3000 | 0,0017 | 0,0076 | snp8\|snp9\|snp10\|snp11\|snp12\|snp13\|snp14\|snp15\|snp16\|snp17\|snp18 |
| H9 | TTAAAGAACAT | 0,1750 | 0,4181 | 0,0173 | 0,0492 | snp8\|snp9\|snp10\|snp11\|snp12\|snp13\|snp14\|snp15\|snp16\|snp17\|snp18 |
| H9 | TCAAAGAACAT | 0,0250 | 0,0000 | 0,3143 | 0,3922 | snp8\|snp9\|snp10\|snp11\|snp12\|snp13\|snp14\|snp15\|snp16\|snp17\|snp18 |
| H9 | TTAAAGAGCAT | 0,0250 | 0,0319 | 0,8520 | 0,7833 | snp8\|snp9\|snp10\|snp11\|snp12\|snp13\|snp14\|snp15\|snp16\|snp17\|snp18 |
| H9 | TCCGTAGGAGT | 0,0500 | 0,0250 | 0,5562 | 0,2709 | snp8\|snp9\|snp10\|snp11\|snp12\|snp13\|snp14\|snp15\|snp16\|snp17\|snp18 |
| H9 | TTAAAAGGAGT | 0,0500 | 0,1500 | 0,1360 | 0,1747 | snp8\|snp9\|snp10\|snp11\|snp12\|snp13\|snp14\|snp15\|snp16\|snp17\|snp18 |
| H9 | TCCGTGGAAGT | 0,0000 | 0,0250 | 0,3143 | 0,1054 | snp8\|snp9\|snp10\|snp11\|snp12\|snp13\|snp14\|snp15\|snp16\|snp17\|snp18 |
| H9 | TCCGTGGGCGC | 0,0000 | 0,0250 | 0,3143 | 0,1054 | snp8\|snp9\|snp10\|snp11\|snp12\|snp13\|snp14\|snp15\|snp16\|snp17\|snp18 |
| H9 | CCCGAGGGCGC | 0,0250 | 0,0000 | 0,3143 | 0,5436 | snp8\|snp9\|snp10\|snp11\|snp12\|snp13\|snp14\|snp15\|snp16\|snp17\|snp18 |
| H10 | CC | 0,6750 | 0,3000 | 0,0008 | 0,0042 | snp8\|snp18 |
| H10 | TC | 0,0000 | 0,0250 | 0,3143 | 0,1054 | snp8\|snp18 |
| H10 | CT | 0,0000 | 0,0250 | 0,3143 | 0,1445 | snp8\|snp18 |
| H10 | TT | 0,3250 | 0,6500 | 0,0036 | 0,0291 | snp8\|snp18 |
| H11 | TT | 0,2500 | 0,6000 | 0,0015 | 0,0256 | snp8\|snp9 |
| H11 | CC | 0,6750 | 0,3250 | 0,0017 | 0,0132 | snp8\|snp9 |
| H11 | TC | 0,0750 | 0,0750 | 1,0000 | 0,8564 | snp8\|snp9 |
| H12 | CCC | 0,6750 | 0,3000 | 0,0008 | 0,0042 | snp8\|snp9\|snp18 |
| H12 | TCC | 0,0000 | 0,0250 | 0,3143 | 0,1054 | snp8\|snp9\|snp18 |
| H12 | TTT | 0,2500 | 0,6000 | 0,0015 | 0,0256 | snp8\|snp9\|snp18 |
| H12 | CCT | 0,0000 | 0,0250 | 0,3143 | 0,1445 | snp8\|snp9\|snp18 |
| H12 | TCT | 0,0750 | 0,0500 | 0,6442 | 0,6020 | snp8\|snp9\|snp18 |
| H13 | CCC | 0,6750 | 0,3000 | 0,0008 | 0,0042 | snp8\|snp10\|snp18 |
| H13 | TCC | 0,0000 | 0,0250 | 0,3143 | 0,1054 | snp8\|snp10\|snp18 |
| H13 | TAT | 0,2750 | 0,6000 | 0,0034 | 0,0367 | snp8\|snp10\|snp18 |
| H13 | CCT | 0,0000 | 0,0250 | 0,3143 | 0,1445 | snp8\|snp10\|snp18 |
| H13 | TCT | 0,0500 | 0,0500 | 1,0000 | 0,8959 | snp8\|snp10\|snp18 |
| H14 | CGC | 0,6750 | 0,3000 | 0,0008 | 0,0042 | snp8\|snp11\|snp18 |
| H14 | TGC | 0,0000 | 0,0250 | 0,3143 | 0,1054 | snp8\|snp11\|snp18 |
| H14 | TAT | 0,2750 | 0,6000 | 0,0034 | 0,0367 | snp8\|snp11\|snp18 |
| H14 | CGT | 0,0000 | 0,0250 | 0,3143 | 0,1445 | snp8\|snp11\|snp18 |
| H14 | TGT | 0,0500 | 0,0500 | 1,0000 | 0,8959 | snp8\|snp11\|snp18 |
| H15 | CAC | 0,0250 | 0,0000 | 0,3143 | 0,5436 | snp8\|snp12\|snp18 |
| H15 | CTC | 0,6500 | 0,3000 | 0,0017 | 0,0076 | snp8\|snp12\|snp18 |
| H15 | TTC | 0,0000 | 0,0250 | 0,3143 | 0,1054 | snp8\|snp12\|snp18 |
| H15 | TAT | 0,2750 | 0,5946 | 0,0039 | 0,0415 | snp8\|snp12\|snp18 |
| H15 | CTT | 0,0000 | 0,0250 | 0,3143 | 0,1445 | snp8\|snp12\|snp18 |
| H15 | TTT | 0,0500 | 0,0554 | 0,9135 | 0,9648 | snp8\|snp12\|snp18 |
| H16 | CGC | 0,6750 | 0,3000 | 0,0008 | 0,0042 | snp8\|snp13\|snp18 |
| H16 | TGC | 0,0000 | 0,0250 | 0,3143 | 0,1054 | snp8\|snp13\|snp18 |
| H16 | TAT | 0,1000 | 0,1899 | 0,2536 | 0,4522 | snp8\|snp13\|snp18 |
| H16 | CGT | 0,0000 | 0,0250 | 0,3143 | 0,1445 | snp8\|snp13\|snp18 |
| H16 | TGT | 0,2250 | 0,4601 | 0,0267 | 0,0251 | snp8\|snp13\|snp18 |
| H17 | CGC | 0,6750 | 0,3000 | 0,0008 | 0,0042 | snp8\|snp14\|snp18 |
| H17 | TGC | 0,0000 | 0,0250 | 0,3143 | 0,1054 | snp8\|snp14\|snp18 |
| H17 | TAT | 0,2250 | 0,4250 | 0,0562 | 0,0799 | snp8\|snp14\|snp18 |
| H17 | CGT | 0,0000 | 0,0250 | 0,3143 | 0,1445 | snp8\|snp14\|snp18 |
| H17 | TGT | 0,1000 | 0,2250 | 0,1297 | 0,1770 | snp8\|snp14\|snp18 |
| H18 | CGC | 0,6750 | 0,3000 | 0,0008 | 0,0042 | snp8\|snp15\|snp18 |
| H18 | TGC | 0,0000 | 0,0250 | 0,3143 | 0,1054 | snp8\|snp15\|snp18 |
| H18 | TAT | 0,2000 | 0,4324 | 0,0254 | 0,0324 | snp8\|snp15\|snp18 |
| H18 | CGT | 0,0000 | 0,0250 | 0,3143 | 0,1445 | snp8\|snp15\|snp18 |
| H18 | TGT | 0,1250 | 0,2176 | 0,2719 | 0,4272 | snp8\|snp15\|snp18 |
| H19 | TAC | 0,0000 | 0,0250 | 0,3143 | 0,1054 | snp8\|snp16\|snp18 |
| H19 | CCC | 0,6750 | 0,3000 | 0,0008 | 0,0042 | snp8\|snp16\|snp18 |
| H19 | TAT | 0,1000 | 0,1899 | 0,2536 | 0,4522 | snp8\|snp16\|snp18 |
| H19 | CCT | 0,0000 | 0,0250 | 0,3143 | 0,1445 | snp8\|snp16\|snp18 |
| H19 | TCT | 0,2250 | 0,4601 | 0,0267 | 0,0251 | snp8\|snp16\|snp18 |
| H20 | CGC | 0,6750 | 0,3000 | 0,0008 | 0,0042 | snp8\|snp17\|snp18 |
| H20 | TGC | 0,0000 | 0,0250 | 0,3143 | 0,1054 | snp8\|snp17\|snp18 |
| H20 | TAT | 0,2171 | 0,4500 | 0,0272 | 0,0553 | snp8\|snp17\|snp18 |
| H20 | CGT | 0,0000 | 0,0250 | 0,3143 | 0,1445 | snp8\|snp17\|snp18 |
| H20 | TGT | 0,1079 | 0,2000 | 0,2537 | 0,3060 | snp8\|snp17\|snp18 |
| H21 | TA | 0,2750 | 0,6000 | 0,0034 | 0,0367 | snp8\|snp10 |
| H21 | CC | 0,6750 | 0,3250 | 0,0017 | 0,0132 | snp8\|snp10 |
| H21 | TC | 0,0500 | 0,0750 | 0,6442 | 0,5996 | snp8\|snp10 |
| H22 | TA | 0,2750 | 0,6000 | 0,0034 | 0,0367 | snp8\|snp11 |
| H22 | CG | 0,6750 | 0,3250 | 0,0017 | 0,0132 | snp8\|snp11 |
| H22 | TG | 0,0500 | 0,0750 | 0,6442 | 0,5996 | snp8\|snp11 |
| H23 | CA | 0,0250 | 0,0000 | 0,3266 | 0,5627 | snp8\|snp12 |
| H23 | TA | 0,2750 | 0,5789 | 0,0066 | 0,0697 | snp8\|snp12 |
| H23 | CT | 0,6500 | 0,3421 | 0,0066 | 0,0394 | snp8\|snp12 |
| H23 | TT | 0,0500 | 0,0790 | 0,6019 | 0,5380 | snp8\|snp12 |
| H24 | TA | 0,1000 | 0,1842 | 0,2855 | 0,5142 | snp8\|snp13 |
| H24 | CG | 0,6750 | 0,3421 | 0,0033 | 0,0204 | snp8\|snp13 |
| H24 | TG | 0,2250 | 0,4737 | 0,0210 | 0,0161 | snp8\|snp13 |
| H25 | TA | 0,2250 | 0,4167 | 0,0727 | 0,1144 | snp8\|snp14 |
| H25 | CG | 0,6750 | 0,3333 | 0,0029 | 0,0221 | snp8\|snp14 |
| H25 | TG | 0,1000 | 0,2500 | 0,0829 | 0,1134 | snp8\|snp14 |
| H26 | TA | 0,2000 | 0,4211 | 0,0345 | 0,0459 | snp8\|snp15 |
| H26 | CG | 0,6750 | 0,3421 | 0,0033 | 0,0204 | snp8\|snp15 |
| H26 | TG | 0,1250 | 0,2368 | 0,1983 | 0,2254 | snp8\|snp15 |
| H27 | TA | 0,1000 | 0,2105 | 0,1763 | 0,2309 | snp8\|snp16 |
| H27 | CC | 0,6750 | 0,3421 | 0,0033 | 0,0204 | snp8\|snp16 |
| H27 | TC | 0,2250 | 0,4474 | 0,0373 | 0,0369 | snp8\|snp16 |
| H28 | TA | 0,2105 | 0,4500 | 0,0249 | 0,0566 | snp8\|snp17 |
| H28 | CG | 0,6842 | 0,3250 | 0,0015 | 0,0138 | snp8\|snp17 |
| H28 | TG | 0,1053 | 0,2250 | 0,1561 | 0,1668 | snp8\|snp17 |
| H29 | TA | 0,2750 | 0,6000 | 0,0034 | 0,0367 | snp18\|snp10 |
| H29 | CC | 0,6750 | 0,3250 | 0,0017 | 0,0107 | snp18\|snp10 |
| H29 | TC | 0,0500 | 0,0750 | 0,6442 | 0,5743 | snp18\|snp10 |
| H30 | TA | 0,2750 | 0,6000 | 0,0034 | 0,0367 | snp18\|snp11 |
| H30 | CG | 0,6750 | 0,3250 | 0,0017 | 0,0107 | snp18\|snp11 |
| H30 | TG | 0,0500 | 0,0750 | 0,6442 | 0,5743 | snp18\|snp11 |
| H31 | CA | 0,0250 | 0,0000 | 0,3266 | 0,5627 | snp18\|snp12 |
| H31 | TA | 0,2750 | 0,5789 | 0,0066 | 0,0697 | snp18\|snp12 |
| H31 | CT | 0,6500 | 0,3421 | 0,0066 | 0,0344 | snp18\|snp12 |
| H31 | TT | 0,0500 | 0,0790 | 0,6019 | 0,5026 | snp18\|snp12 |
| H32 | TA | 0,1000 | 0,1842 | 0,2855 | 0,5142 | snp18\|snp13 |
| H32 | CG | 0,6750 | 0,3421 | 0,0033 | 0,0168 | snp18\|snp13 |
| H32 | TG | 0,2250 | 0,4737 | 0,0210 | 0,0111 | snp18\|snp13 |
| H33 | TA | 0,2250 | 0,4167 | 0,0727 | 0,1144 | snp18\|snp14 |
| H33 | CG | 0,6750 | 0,3333 | 0,0029 | 0,0183 | snp18\|snp14 |
| H33 | TG | 0,1000 | 0,2500 | 0,0829 | 0,0869 | snp18\|snp14 |
| H34 | TA | 0,2000 | 0,4211 | 0,0345 | 0,0459 | snp18\|snp15 |
| H34 | CG | 0,6750 | 0,3421 | 0,0033 | 0,0168 | snp18\|snp15 |
| H34 | TG | 0,1250 | 0,2368 | 0,1983 | 0,2458 | snp18\|snp15 |
| H35 | TA | 0,1000 | 0,2105 | 0,1763 | 0,2309 | snp18\|snp16 |
| H35 | CC | 0,6750 | 0,3421 | 0,0033 | 0,0168 | snp18\|snp16 |
| H35 | TC | 0,2250 | 0,4474 | 0,0373 | 0,0410 | snp18\|snp16 |
| H36 | TA | 0,2105 | 0,4500 | 0,0249 | 0,0566 | snp18\|snp17 |
| H36 | CG | 0,6842 | 0,3250 | 0,0015 | 0,0112 | snp18\|snp17 |
| H36 | TG | 0,1053 | 0,2250 | 0,1561 | 0,1374 | snp18\|snp17 |
